# 2,6-Di­amino-4-(4-chloro­phen­yl)-1-methyl-1,4-di­hydro­pyridine-3,5-dicarbo­nitrile

**DOI:** 10.1107/S1600536814014354

**Published:** 2014-06-25

**Authors:** Michael Purushothaman, Kaliyaperumal Thanigaimani, Suhana Arshad, Sekar Silambarasan, Ibrahim Abdul Razak, Kather Mohideen Sithick Ali

**Affiliations:** a Department of Chemistry, Jamal Mohamed College (Autonomous), Tiruchirappalli 620 020, Tamil Nadu, India; b School of Physics, Universiti Sains Malaysia, 11800 USM, Penang, Malaysia

**Keywords:** crystal structure

## Abstract

In the title compound, C_14_H_12_ClN_5_, the di­hydro­pyridine ring adopts a shallow boat conformation. The dihedral angle between the plane of this ring and that of the chloro­benzene ring is 69.15 (15)°. In the crystal, mol­ecules are linked by N—H⋯N and N—H⋯Cl hydrogen bonds, generating (001) sheets.

## Related literature   

For background to malono­nitrile, see: Fatiadi (1978 ▶); Raghukumar *et al.* (2003 ▶). For the stability of the temperature controller used for the data collection, see: Cosier & Glazer (1986 ▶).
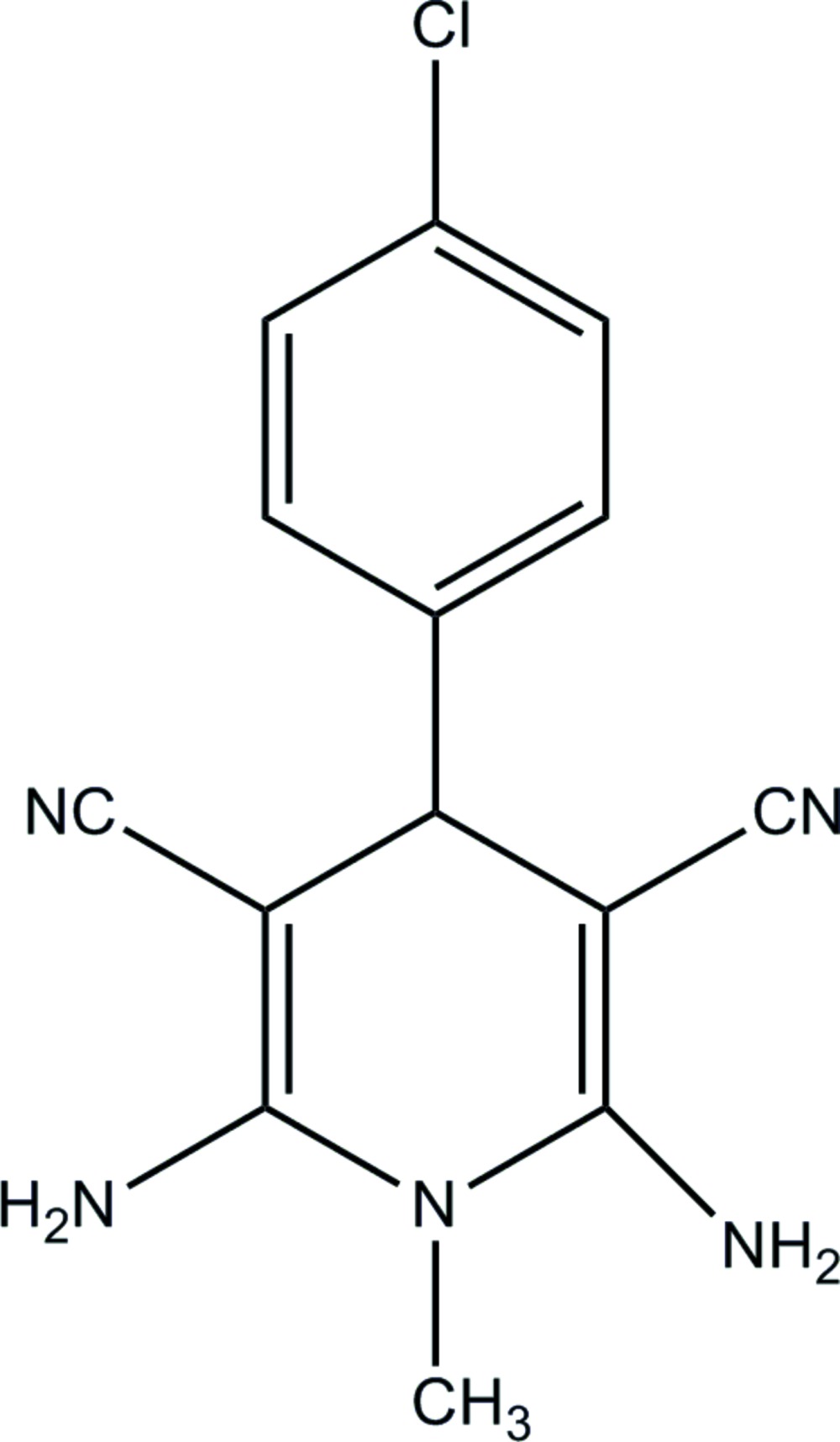


## Experimental   

### 

#### Crystal data  

C_14_H_12_ClN_5_
*M* *_r_* = 285.74
Triclinic, 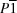

*a* = 8.3893 (4) Å
*b* = 8.4679 (5) Å
*c* = 10.2571 (6) Å
α = 93.148 (4)°
β = 112.478 (3)°
γ = 93.929 (3)°
*V* = 669.11 (6) Å^3^
*Z* = 2
Mo *K*α radiation
μ = 0.28 mm^−1^
*T* = 100 K
0.37 × 0.28 × 0.16 mm

#### Data collection  

Bruker SMART APEXII CCD diffractometer
Absorption correction: multi-scan (*SADABS*; Bruker, 2009 ▶) *T*_min_ = 0.904, *T*_max_ = 0.956
9775 measured reflections
3049 independent reflections
2435 reflections with *I* > 2σ(*I*)
*R*_int_ = 0.053

#### Refinement  

*R*[*F* ^2^ > 2σ(*F* ^2^)] = 0.072
*wR*(*F* ^2^) = 0.207
*S* = 1.05
3049 reflections
198 parameters
H atoms treated by a mixture of independent and constrained refinement
Δρ_max_ = 1.17 e Å^−3^
Δρ_min_ = −0.48 e Å^−3^

### 

Data collection: *APEX2* (Bruker, 2009 ▶); cell refinement: *SAINT* (Bruker, 2009 ▶); data reduction: *SAINT*; program(s) used to solve structure: *SHELXTL* (Sheldrick, 2008 ▶); program(s) used to refine structure: *SHELXTL*; molecular graphics: *SHELXTL*; software used to prepare material for publication: *SHELXTL* and *PLATON* (Spek, 2009 ▶).

## Supplementary Material

Crystal structure: contains datablock(s) global, I. DOI: 10.1107/S1600536814014354/hb7240sup1.cif

Structure factors: contains datablock(s) I. DOI: 10.1107/S1600536814014354/hb7240Isup2.hkl

Supporting information file. DOI: 10.1107/S1600536814014354/hb7240Isup3.cml

CCDC reference: 1009061

Additional supporting information:  crystallographic information; 3D view; checkCIF report

## Figures and Tables

**Table 1:** Hydrogen-bond geometry (Å, °)

*D*—H⋯*A*	*D*—H	H⋯*A*	*D*⋯*A*	*D*—H⋯*A*
N2—H1*N*2⋯N5^i^	0.86 (5)	2.21 (5)	3.043 (4)	163 (5)
N2—H2*N*2⋯Cl1^ii^	0.89 (4)	2.75 (4)	3.588 (3)	158 (3)
N3—H2*N*3⋯N4^iii^	0.83 (4)	2.27 (4)	3.071 (4)	161 (4)

Symmetry codes: (i) 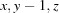; (ii) 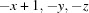; (iii) 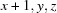.

**Fig. 1.**
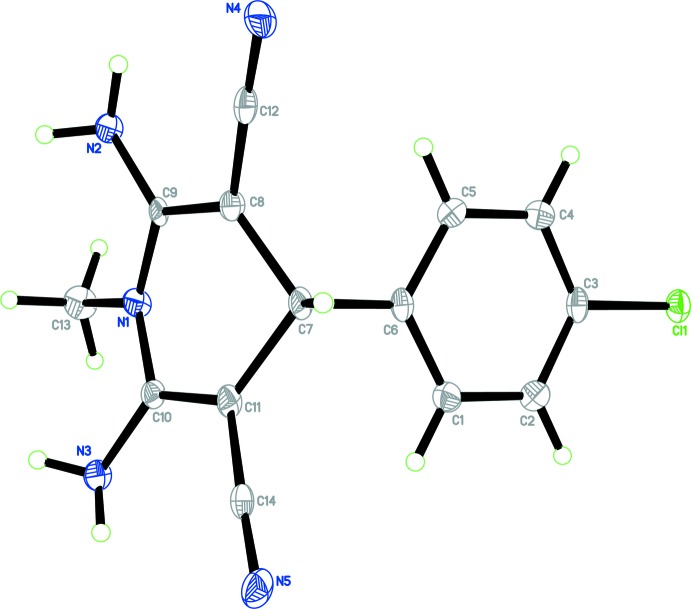
The molecular structure of the title compound with 50% probability displacement ellipsoids.

**Fig. 2.**
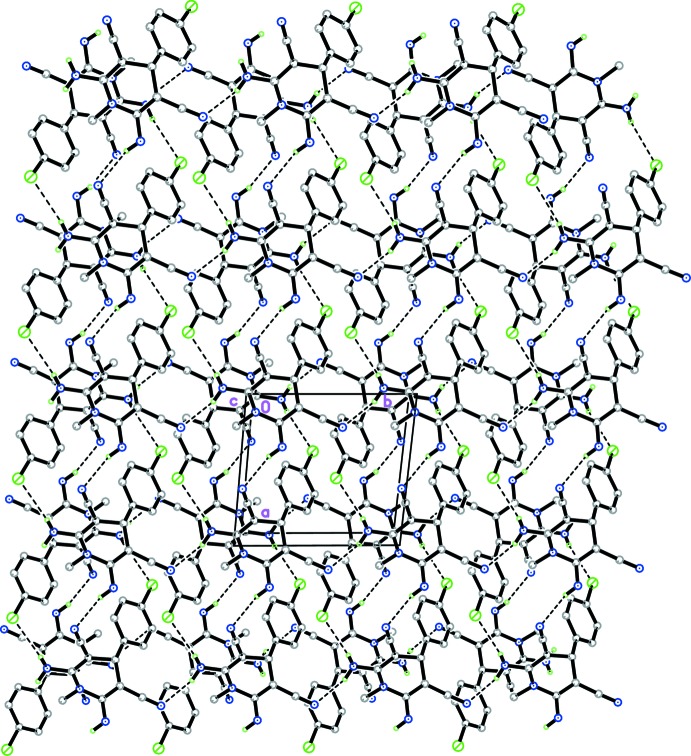
The crystal packing of the title compound. The H atoms not involved in the intermolecular interactions (dashed lines) have been omitted for clarity.

## supplementary crystallographic information

### S1. Comment

Malononitrile is a simple and versatile reagent for the synthesis of heterocyclic compounds and precursors of novel compounds. It exhibits a unique reactivity due to the strong electron withdrawing cycano groups to activate the methylene group and the polar multiple bond suitable for nucleophilic addition (Fatiadi, 1978). Malononitrile is used as reactant or reaction intermediate in various multicomponent reactions to prepare heterocyclic compounds. The three component reactions of malononitrile, aldehyde and amine show very chemical diversity, from which several kinds of products were separated (Raghukumar *et al.*, 2003). The crystal structure of the title compound (I) is presented here.

The molecular structure of the title compound is shown in Fig. 1. The pyridine ring (N1/C7—C11) adopts a boat conformation with puckering parameters Q= 0.402 (3) Å, Θ= 79.6 (4)° and Φ= 177.2 (4)°. The dihedral angle between the pyridine (N1/C7—C11) and benzene (C1—C6) rings is 69.15 (15) °.

The crystal structure shown in Fig. 2 features N2—H1N2···N5^i^ and N3—H2N3···N4^iii^ hydrogen bonds (symmetry code in Table 1) to result in tetrameric association of molecules, generated by inversion. These tetramers are then connected *via* N2—H2N2···Cl1^ii^ hydrogen bond (symmetry code in Table 1), forming a layer parallel to the *ab* plane.

### S2. Experimental

Compound (I) was prepared by the reaction of *p*-chlorobenzaldehyde (1 mmol), malononitrile (1 mmol) and methylamine (1 mmol) in a mixed solvent of methanol and water (5:1) was stirred at room temperature about an hour. The resulting precipitate was collected by filtration and washed with methanol to afford pure product, *m.p*: 290 °C. The product was crystallized from methanol solution as colourless plates.

### S3. Refinement

N-bound H atoms were located in a difference Fourier maps and allowed to be refined freely [refined distance: N–H = 0.83 (4)–0.89 (4) Å]. The remaining hydrogen atoms were positioned geometrically [C–H= 0.95 or 0.98 Å] and were refined using a riding model, with *U*_iso_(H) = 1.2 *U*_eq_(C) or 1.5 *U*_eq_(methyl C). A rotating-group model was used for the methyl group.

### Crystal data

**Table:**

C_14_H_12_ClN_5_	*Z* = 2
*M**_r_* = 285.74	*F*(000) = 296
Triclinic, *P*1	*D*_x_ = 1.418 Mg m^−^^3^
Hall symbol: -P 1	Mo *K*α radiation, λ = 0.71073 Å
*a* = 8.3893 (4) Å	Cell parameters from 4444 reflections
*b* = 8.4679 (5) Å	θ = 2.6–32.3°
*c* = 10.2571 (6) Å	µ = 0.28 mm^−^^1^
α = 93.148 (4)°	*T* = 100 K
β = 112.478 (3)°	Plate, colourless
γ = 93.929 (3)°	0.37 × 0.28 × 0.16 mm
*V* = 669.11 (6) Å^3^	

### Data collection

**Table:**

Bruker SMART APEXII CCD diffractometer	3049 independent reflections
Radiation source: fine-focus sealed tube	2435 reflections with *I* > 2σ(*I*)
Graphite monochromator	*R*_int_ = 0.053
φ and ω scans	θ_max_ = 27.5°, θ_min_ = 2.2°
Absorption correction: multi-scan (*SADABS*; Bruker, 2009)	*h* = −10→10
*T*_min_ = 0.904, *T*_max_ = 0.956	*k* = −10→9
9775 measured reflections	*l* = −13→13

### Refinement

**Table:**

Refinement on *F*^2^	Primary atom site location: structure-invariant direct methods
Least-squares matrix: full	Secondary atom site location: difference Fourier map
*R*[*F*^2^ > 2σ(*F*^2^)] = 0.072	Hydrogen site location: inferred from neighbouring sites
*wR*(*F*^2^) = 0.207	H atoms treated by a mixture of independent and constrained refinement
*S* = 1.05	*w* = 1/[σ^2^(*F*_o_^2^) + (0.1391*P*)^2^ + 0.3591*P*] where *P* = (*F*_o_^2^ + 2*F*_c_^2^)/3
3049 reflections	(Δ/σ)_max_ < 0.001
198 parameters	Δρ_max_ = 1.17 e Å^−^^3^
0 restraints	Δρ_min_ = −0.48 e Å^−^^3^

### Special details

**Table:**

Experimental. The crystal was placed in the cold stream of an Oxford Cryosystems Cobra open-flow nitrogen cryostat (Cosier & Glazer, 1986) operating at 100.0 (1) K.
Geometry. All e.s.d.'s (except the e.s.d. in the dihedral angle between two l.s. planes) are estimated using the full covariance matrix. The cell e.s.d.'s are taken into account individually in the estimation of e.s.d.'s in distances, angles and torsion angles; correlations between e.s.d.'s in cell parameters are only used when they are defined by crystal symmetry. An approximate (isotropic) treatment of cell e.s.d.'s is used for estimating e.s.d.'s involving l.s. planes.
Refinement. Refinement of *F*^2^ against ALL reflections. The weighted *R*-factor *wR* and goodness of fit *S* are based on *F*^2^, conventional *R*-factors *R* are based on *F*, with *F* set to zero for negative *F*^2^. The threshold expression of *F*^2^ > σ(*F*^2^) is used only for calculating *R*-factors(gt) *etc*. and is not relevant to the choice of reflections for refinement. *R*-factors based on *F*^2^ are statistically about twice as large as those based on *F*, and *R*- factors based on ALL data will be even larger.

### Fractional atomic coordinates and isotropic or equivalent isotropic displacement parameters (Å^2^)

**Table:**

	*x*	*y*	*z*	*U*_iso_*/*U*_eq_	
Cl1	0.41603 (9)	0.41701 (9)	−0.28944 (7)	0.0186 (3)	
N1	1.1252 (3)	0.0360 (3)	0.2661 (3)	0.0153 (5)	
N2	0.9603 (3)	−0.1777 (3)	0.3020 (3)	0.0164 (5)	
N3	1.3641 (3)	0.2241 (3)	0.3245 (3)	0.0171 (6)	
N4	0.6529 (3)	0.0004 (3)	0.4178 (3)	0.0184 (6)	
N5	1.2267 (3)	0.5888 (3)	0.4289 (3)	0.0198 (6)	
C1	0.8304 (4)	0.4173 (4)	0.0726 (3)	0.0181 (6)	
H1A	0.9409	0.4758	0.1176	0.022*	
C2	0.7184 (4)	0.4557 (4)	−0.0597 (3)	0.0189 (6)	
H2A	0.7516	0.5389	−0.1053	0.023*	
C3	0.5572 (4)	0.3693 (4)	−0.1231 (3)	0.0157 (6)	
C4	0.5057 (4)	0.2498 (4)	−0.0585 (3)	0.0187 (6)	
H4A	0.3943	0.1930	−0.1031	0.022*	
C5	0.6194 (4)	0.2132 (4)	0.0734 (3)	0.0182 (6)	
H5A	0.5844	0.1308	0.1188	0.022*	
C6	0.7834 (3)	0.2950 (3)	0.1401 (3)	0.0136 (6)	
C7	0.9013 (3)	0.2604 (3)	0.2911 (3)	0.0133 (6)	
H7A	0.8677	0.3263	0.3587	0.016*	
C8	0.8816 (4)	0.0898 (3)	0.3211 (3)	0.0137 (6)	
C9	0.9872 (3)	−0.0158 (3)	0.2979 (3)	0.0110 (6)	
C10	1.1955 (4)	0.1912 (3)	0.3081 (3)	0.0135 (6)	
C11	1.0935 (3)	0.3048 (3)	0.3278 (3)	0.0141 (6)	
C12	0.7568 (4)	0.0383 (3)	0.3743 (3)	0.0153 (6)	
C13	1.2207 (4)	−0.0783 (4)	0.2184 (3)	0.0200 (7)	
H13A	1.1391	−0.1654	0.1568	0.030*	
H13B	1.3055	−0.1210	0.3008	0.030*	
H13C	1.2811	−0.0245	0.1657	0.030*	
C14	1.1690 (4)	0.4592 (4)	0.3845 (3)	0.0145 (6)	
H1N2	1.050 (6)	−0.226 (6)	0.345 (5)	0.043 (12)*	
H2N2	0.873 (5)	−0.220 (5)	0.322 (4)	0.022 (9)*	
H1N3	1.414 (5)	0.315 (6)	0.355 (4)	0.030 (11)*	
H2N3	1.425 (5)	0.150 (5)	0.355 (4)	0.029 (11)*	

### Atomic displacement parameters (Å^2^)

**Table:**

	*U*^11^	*U*^22^	*U*^33^	*U*^12^	*U*^13^	*U*^23^
Cl1	0.0168 (4)	0.0188 (4)	0.0178 (4)	0.0060 (3)	0.0030 (3)	0.0034 (3)
N1	0.0126 (11)	0.0132 (12)	0.0215 (12)	0.0042 (9)	0.0080 (10)	−0.0010 (10)
N2	0.0137 (12)	0.0082 (12)	0.0282 (14)	0.0055 (10)	0.0084 (11)	0.0015 (10)
N3	0.0126 (12)	0.0132 (13)	0.0292 (14)	0.0081 (11)	0.0107 (11)	0.0041 (11)
N4	0.0145 (12)	0.0168 (13)	0.0261 (14)	0.0081 (10)	0.0090 (11)	0.0030 (11)
N5	0.0210 (13)	0.0136 (13)	0.0244 (13)	0.0080 (10)	0.0074 (11)	0.0020 (10)
C1	0.0150 (13)	0.0141 (14)	0.0235 (15)	0.0024 (11)	0.0056 (12)	−0.0009 (12)
C2	0.0189 (14)	0.0164 (15)	0.0226 (15)	0.0036 (12)	0.0088 (12)	0.0034 (12)
C3	0.0175 (13)	0.0154 (14)	0.0153 (13)	0.0113 (11)	0.0058 (11)	0.0010 (11)
C4	0.0156 (13)	0.0162 (15)	0.0232 (15)	0.0019 (11)	0.0062 (12)	0.0023 (12)
C5	0.0178 (14)	0.0135 (14)	0.0232 (15)	0.0020 (11)	0.0074 (12)	0.0048 (12)
C6	0.0139 (13)	0.0130 (14)	0.0168 (13)	0.0094 (11)	0.0081 (11)	0.0002 (11)
C7	0.0117 (12)	0.0118 (14)	0.0173 (14)	0.0068 (10)	0.0057 (11)	0.0005 (11)
C8	0.0131 (12)	0.0112 (13)	0.0189 (14)	0.0056 (11)	0.0080 (11)	0.0016 (11)
C9	0.0092 (12)	0.0084 (13)	0.0146 (13)	0.0072 (10)	0.0028 (10)	−0.0003 (10)
C10	0.0124 (13)	0.0136 (14)	0.0160 (13)	0.0054 (11)	0.0064 (11)	0.0022 (11)
C11	0.0129 (13)	0.0131 (14)	0.0174 (14)	0.0064 (11)	0.0065 (11)	0.0003 (11)
C12	0.0151 (13)	0.0130 (14)	0.0167 (14)	0.0091 (11)	0.0036 (11)	0.0022 (11)
C13	0.0195 (14)	0.0142 (15)	0.0297 (17)	0.0075 (12)	0.0130 (13)	−0.0041 (12)
C14	0.0114 (12)	0.0164 (15)	0.0160 (13)	0.0086 (11)	0.0041 (11)	0.0032 (11)

### Geometric parameters (Å, º)

**Table:**

Cl1—C3	1.753 (3)	C3—C4	1.372 (4)
N1—C9	1.369 (4)	C4—C5	1.391 (4)
N1—C10	1.378 (4)	C4—H4A	0.9500
N1—C13	1.474 (4)	C5—C6	1.392 (4)
N2—C9	1.379 (4)	C5—H5A	0.9500
N2—H1N2	0.86 (5)	C6—C7	1.543 (4)
N2—H2N2	0.89 (4)	C7—C8	1.505 (4)
N3—C10	1.366 (4)	C7—C11	1.523 (4)
N3—H1N3	0.83 (5)	C7—H7A	1.0000
N3—H2N3	0.83 (4)	C8—C9	1.373 (4)
N4—C12	1.155 (4)	C8—C12	1.410 (4)
N5—C14	1.162 (4)	C10—C11	1.386 (4)
C1—C6	1.393 (4)	C11—C14	1.403 (4)
C1—C2	1.395 (4)	C13—H13A	0.9800
C1—H1A	0.9500	C13—H13B	0.9800
C2—C3	1.388 (4)	C13—H13C	0.9800
C2—H2A	0.9500		
			
C9—N1—C10	118.3 (2)	C8—C7—C11	107.0 (2)
C9—N1—C13	120.6 (2)	C8—C7—C6	113.9 (2)
C10—N1—C13	119.7 (2)	C11—C7—C6	113.9 (2)
C9—N2—H1N2	117 (3)	C8—C7—H7A	107.2
C9—N2—H2N2	121 (3)	C11—C7—H7A	107.2
H1N2—N2—H2N2	108 (4)	C6—C7—H7A	107.2
C10—N3—H1N3	120 (3)	C9—C8—C12	119.9 (3)
C10—N3—H2N3	113 (3)	C9—C8—C7	119.8 (3)
H1N3—N3—H2N3	115 (4)	C12—C8—C7	120.3 (2)
C6—C1—C2	121.3 (3)	C8—C9—N1	120.7 (3)
C6—C1—H1A	119.4	C8—C9—N2	123.1 (3)
C2—C1—H1A	119.4	N1—C9—N2	116.2 (2)
C3—C2—C1	118.4 (3)	N3—C10—N1	116.7 (3)
C3—C2—H2A	120.8	N3—C10—C11	123.7 (3)
C1—C2—H2A	120.8	N1—C10—C11	119.6 (3)
C4—C3—C2	121.8 (3)	C10—C11—C14	119.7 (3)
C4—C3—Cl1	119.6 (2)	C10—C11—C7	119.9 (3)
C2—C3—Cl1	118.5 (2)	C14—C11—C7	120.4 (2)
C3—C4—C5	118.9 (3)	N4—C12—C8	178.0 (3)
C3—C4—H4A	120.5	N1—C13—H13A	109.5
C5—C4—H4A	120.5	N1—C13—H13B	109.5
C4—C5—C6	121.3 (3)	H13A—C13—H13B	109.5
C4—C5—H5A	119.3	N1—C13—H13C	109.5
C6—C5—H5A	119.3	H13A—C13—H13C	109.5
C1—C6—C5	118.2 (3)	H13B—C13—H13C	109.5
C1—C6—C7	121.4 (3)	N5—C14—C11	177.8 (3)
C5—C6—C7	120.1 (3)		
			
C6—C1—C2—C3	0.3 (5)	C7—C8—C9—N1	8.9 (4)
C1—C2—C3—C4	0.7 (5)	C12—C8—C9—N2	10.8 (4)
C1—C2—C3—Cl1	−180.0 (2)	C7—C8—C9—N2	−169.8 (3)
C2—C3—C4—C5	−0.8 (5)	C10—N1—C9—C8	23.8 (4)
Cl1—C3—C4—C5	179.9 (2)	C13—N1—C9—C8	−169.9 (3)
C3—C4—C5—C6	−0.2 (5)	C10—N1—C9—N2	−157.4 (3)
C2—C1—C6—C5	−1.3 (4)	C13—N1—C9—N2	8.9 (4)
C2—C1—C6—C7	−175.4 (3)	C9—N1—C10—N3	156.2 (3)
C4—C5—C6—C1	1.2 (4)	C13—N1—C10—N3	−10.2 (4)
C4—C5—C6—C7	175.4 (3)	C9—N1—C10—C11	−25.5 (4)
C1—C6—C7—C8	−152.0 (3)	C13—N1—C10—C11	168.1 (3)
C5—C6—C7—C8	34.0 (3)	N3—C10—C11—C14	−8.3 (4)
C1—C6—C7—C11	−28.9 (4)	N1—C10—C11—C14	173.5 (3)
C5—C6—C7—C11	157.1 (3)	N3—C10—C11—C7	173.1 (3)
C11—C7—C8—C9	−34.8 (3)	N1—C10—C11—C7	−5.1 (4)
C6—C7—C8—C9	92.0 (3)	C8—C7—C11—C10	32.9 (4)
C11—C7—C8—C12	144.5 (3)	C6—C7—C11—C10	−93.9 (3)
C6—C7—C8—C12	−88.7 (3)	C8—C7—C11—C14	−145.7 (3)
C12—C8—C9—N1	−170.4 (3)	C6—C7—C11—C14	87.6 (3)

### Hydrogen-bond geometry (Å, º)

**Table:**

*D*—H···*A*	*D*—H	H···*A*	*D*···*A*	*D*—H···*A*
N2—H1*N*2···N5^i^	0.86 (5)	2.21 (5)	3.043 (4)	163 (5)
N2—H2*N*2···Cl1^ii^	0.89 (4)	2.75 (4)	3.588 (3)	158 (3)
N3—H2*N*3···N4^iii^	0.83 (4)	2.27 (4)	3.071 (4)	161 (4)

Symmetry codes: (i) *x*, *y*−1, *z*; (ii) −*x*+1, −*y*, −*z*; (iii) *x*+1, *y*, *z*.
